# Endoscopic Papillectomy for Ampullary Adenoma Adjacent to a Cystic Lesion

**DOI:** 10.7759/cureus.81318

**Published:** 2025-03-27

**Authors:** Koji Takahashi, Kenjiro Yamamoto, Takanori Aihara, Izumi Ohno, Hiroshi Ohyama

**Affiliations:** 1 Gastroenterology, Chiba University, Chiba, JPN; 2 Medical Oncology, Chiba University, Chiba, JPN; 3 Gastroenterology and Hepatology, Tokyo Medical University, Tokyo, JPN; 4 Pathology, Chiba University, Chiba, JPN

**Keywords:** ampulla of the vater, ampullary adenoma, cysts, duodenal adenoma, endoscopy

## Abstract

Ampullary adenomas are precancerous lesions with a high risk of malignant transformation. Endoscopic papillectomy is the standard treatment, but the presence of adjacent cystic lesions poses a technical challenge. An 83-year-old man underwent an endoscopic examination, which revealed an ampullary tumor adjacent to a cystic lesion. Endoscopic ultrasound and MRI confirmed its cystic nature. A biopsy diagnosed an intestinal-type adenoma. The patient underwent en bloc endoscopic papillectomy with simultaneous resection of the cystic lesion. The procedure was complication free, and histopathological analysis confirmed a nonmalignant adenoma with negative resection margins. The cystic lesion was identified as a dilated glandular duct without adenomatous components. This case underscores the importance of detailed imaging for preoperative planning. En bloc endoscopic papillectomy is a feasible and safe approach for ampullary adenomas with adjacent cystic lesions. A thorough preoperative assessment and meticulous procedural planning are crucial for achieving complete resection with negative margins.

## Introduction

Ampullary adenomas can arise sporadically or in association with genetic disorders such as familial adenomatous polyposis [1]. Compared to nonampullary duodenal adenomas, they have a higher risk of malignant transformation, with approximately 30% of untreated cases progressing to adenocarcinoma. However, a long-term follow-up study of 24 sporadic ampullary adenomas that underwent appropriate endoscopic resection found no cases of progression to duodenal papillary cancer [2]. The standard first-line treatment for ampullary adenomas is endoscopic snare papillectomy [3]. While cystic components have been reported in nonampullary duodenal adenomas [4,5], such features remain rare in ampullary adenomas. Here, we present a case of successful endoscopic papillectomy for an ampullary adenoma adjacent to a cystic lesion.

## Case presentation

An 83-year-old man underwent an upper GI endoscopy, which revealed an ampullary tumor. He was referred to our hospital for further evaluation.

Four years earlier, during routine cancer screening, he had been diagnosed with early-stage gastric cancer, confirmed as adenocarcinoma by biopsy, and successfully treated with endoscopic submucosal dissection. In the same year, a screening colonoscopy detected a sigmoid colon polyp, which was initially removed endoscopically. However, histopathological examination revealed deep submucosal invasion by adenocarcinoma, prompting a laparoscopic sigmoidectomy. Final pathology confirmed submucosal invasion without lymph node metastasis (T1N0M0, Stage I, UICC TNM classification, 8th edition), and no adjuvant chemotherapy was administered. Genetic testing for these tumors was not performed.

**Figure 1:**
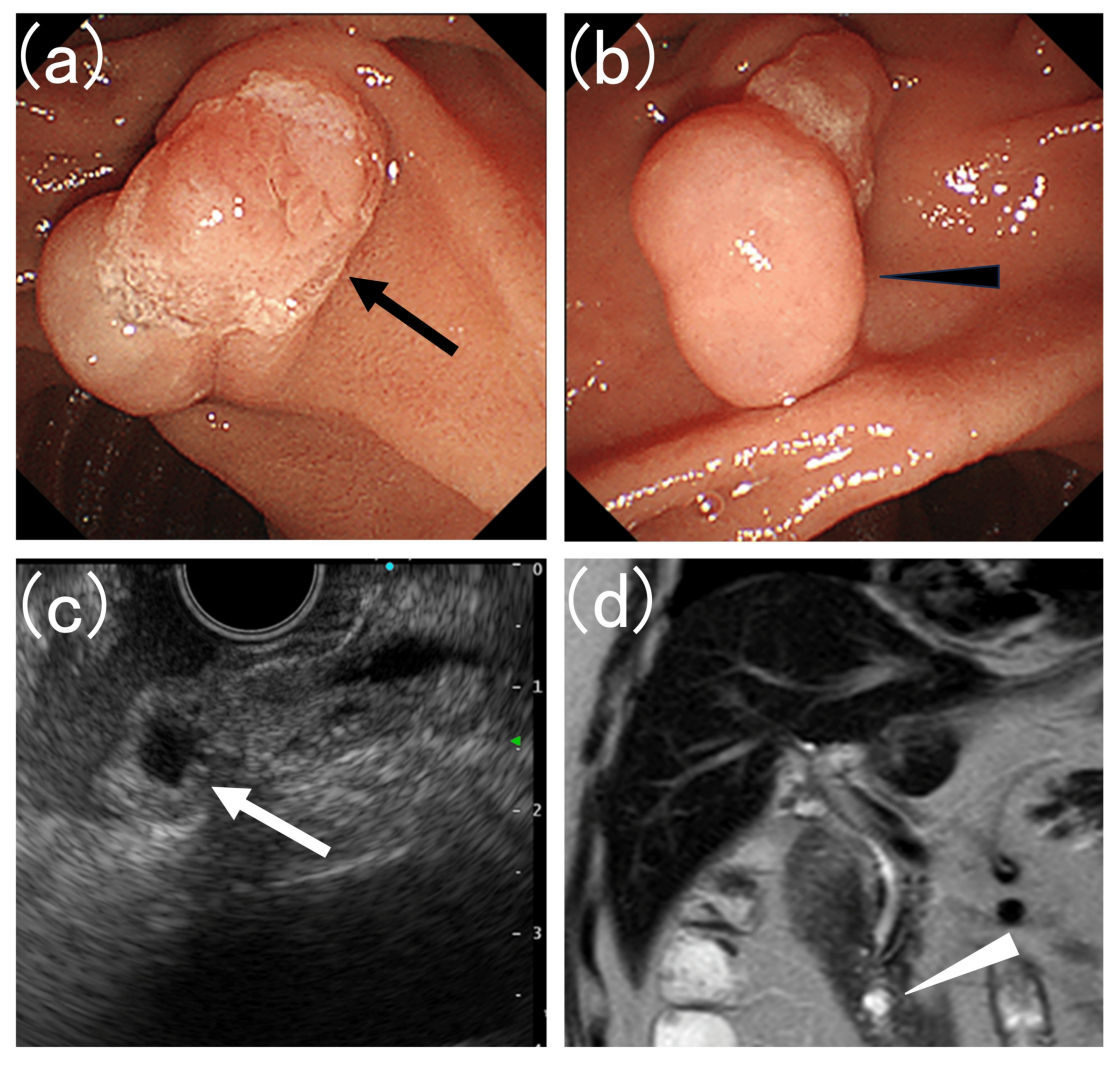
Images of an ampullary adenoma with cystic components (a) Endoscopic examination with a duodenoscope revealed a duodenal papillary tumor (black arrow), and biopsy confirmed the lesion as an adenoma. (b) The ampullary adenoma was associated with a submucosal mass extending toward the anal side (black arrowhead). (c) EUS showed the submucosal mass on the anal side of the ampullary adenoma to be internally anechoic, indicating a cystic nature (white arrow). (d) A coronal T2-weighted magnetic resonance image revealed that the submucosal mass contained only fluid components (white arrowhead), further suggesting it was a cyst. EUS, endoscopic ultrasound

Since then, he has been under regular surveillance with upper GI endoscopy for follow-up of early gastric cancer. During one such follow-up, an enlarged major duodenal papilla was observed. A biopsy confirmed an intestinal-type adenoma. Further evaluation with endoscopic ultrasound (EUS) and MRI identified a submucosal lesion adjacent to the ampulla, consistent with a cystic structure (Figure 1). Although the major duodenal papilla was visualized, the exact location of the adenomatous component could not be clearly identified on imaging.

His medical history also included deep vein thrombosis (DVT) of the lower extremities, diagnosed nine years earlier, for which he was receiving oral edoxaban. At the time of diagnosis, no specific risk factors for DVT had been identified. As part of DVT management, an inferior vena cava filter was also placed. He had no family history of GI cancers or ampullary adenomas.

A biopsy confirmed an intestinal-type adenoma. Based on these findings, endoscopic papillectomy, including resection of the adjacent cyst, was performed to achieve negative resection margins.

On the day of the procedure, oral edoxaban was discontinued. Using an oblique-viewing duodenoscope (TJF-Q290V, Olympus, Tokyo, Japan), en bloc resection - including the cyst - was successfully performed with a 15-mm snare (SnareMaster Plus, Olympus, Tokyo, Japan). The resection site was secured with clips, and plastic stents were placed in both the bile and pancreatic ducts to prevent complications. Oral edoxaban was resumed the following day. The patient experienced no complications related to the endoscopic treatment and was discharged on postoperative day 7 after an uneventful recovery.

**Figure 2:**
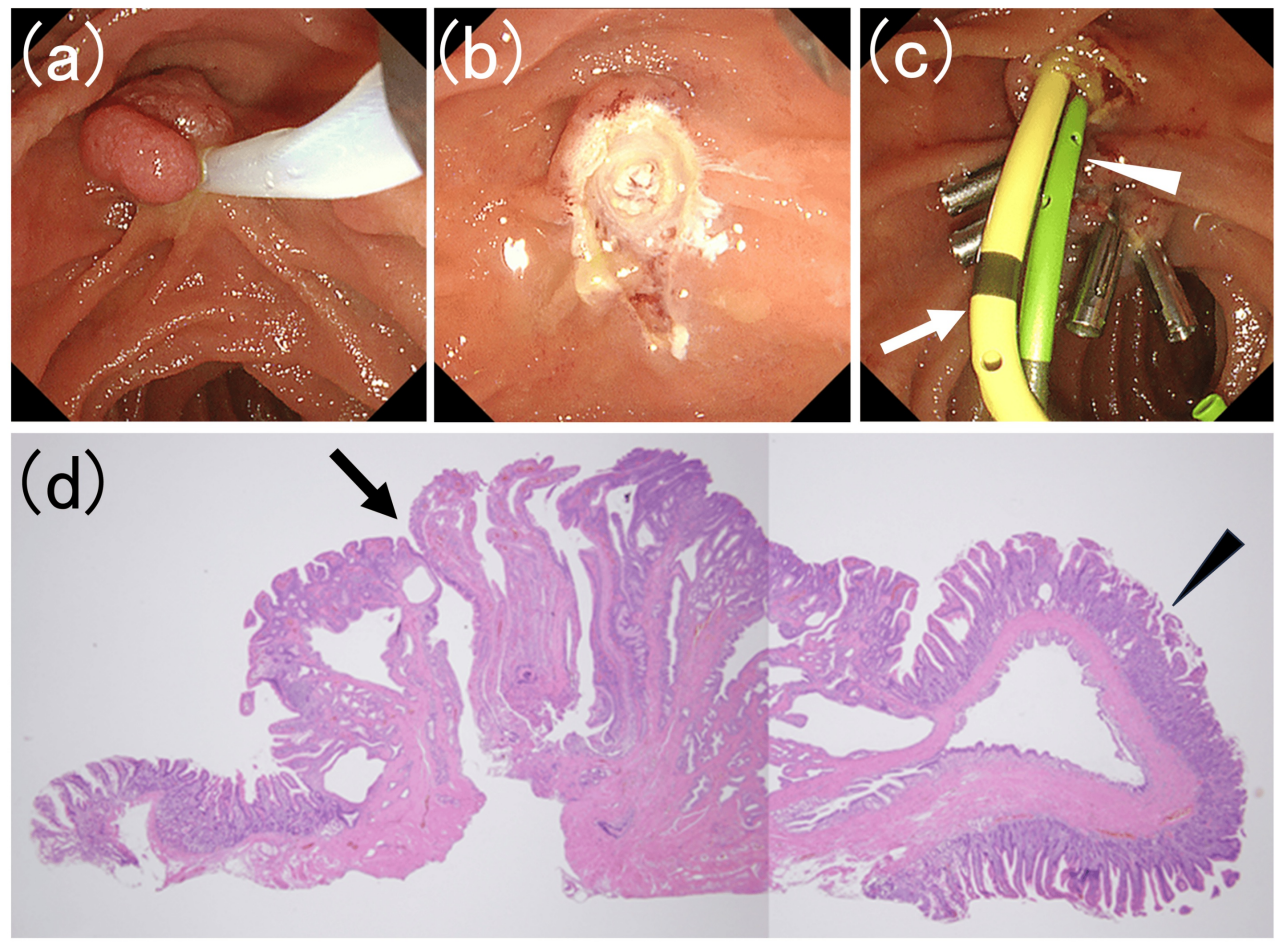
Endoscopic images of the papillectomy procedure for ampullary adenoma and histopathological analysis of the resected specimen (a) The cystic component on the anal side of the ampullary adenoma was resected using a snare. (b) The resection site showed no evidence of residual lesions. (c) The site was secured with clips, and a biliary plastic stent (white arrow) and a pancreatic plastic stent (white arrowhead) were placed to prevent complications. (d) The histopathological evaluation confirmed an intestinal-type adenoma with negative horizontal and vertical margins. The black arrow indicates the biliary orifice. No malignant features were observed. The adjacent cyst (black arrowhead) was identified as a cystic dilated glandular duct without adenomatous components.

Histopathological examination confirmed a nonmalignant intestinal-type adenoma with negative horizontal and vertical margins. The adjacent cyst was identified as a cystically dilated glandular duct with no adenomatous components (Figure 2).

Endoscopic removal of both the biliary and pancreatic duct stents was performed 52 days after tumor resection. At the time of stent removal, endoscopic inspection of the resection scar showed no signs of adenoma recurrence. Biopsy specimens from the scar also revealed no evidence of recurrence. Additionally, no stenosis was observed at either the biliary or pancreatic duct orifice. The patient has remained well without complications since then.

## Discussion

Endoscopic papillectomy is a well-established, minimally invasive alternative to surgical resection for ampullary adenomas [6-8]. It is recommended for lesions smaller than 5 cm without intraductal extension into the bile or pancreatic ducts and with no evidence of malignancy [6,7]. Recent guidelines and large cohort studies have confirmed that endoscopic resection offers high curative rates, along with lower complication rates and shorter hospital stays compared to surgery [8,9].

Despite its efficacy, endoscopic papillectomy requires careful patient selection and long-term follow-up due to the risk of recurrence and complications [10]. Reported curative resection rates range from 76.4% to 80.9%, with complete resection rates between 93.4% and 98.2% [10,11]. However, recurrence occurs in up to 20-30% of cases, with piecemeal resection and positive or indeterminate margins identified as major risk factors [7,10,12]. Late recurrences, occurring up to seven to eight years post-procedure, underscore the need for long-term endoscopic surveillance of at least five years [12,13]. Complications such as pancreatitis, bleeding, perforation, and papillary stenosis are reported in approximately 24.5-30% of cases [6-8]. To reduce the risk of post-procedural pancreatitis and pancreatic duct stenosis, prophylactic placement of a pancreatic duct stent is recommended [7-9,14]. Recent meta-analyses suggest that pancreatic stents, particularly when combined with rectal NSAIDs, significantly lower the risk of pancreatitis [14].

In our case, the presence of a cystic lesion at the distal margin of the ampullary adenoma posed a technical challenge for endoscopic resection. By using a large snare to encompass the cystic portion, we successfully achieved en bloc resection with negative margins. No intraoperative or postoperative complications, such as bleeding or duodenal perforation, were observed. This case highlights the importance of thorough preoperative imaging for procedural planning. Initially suspected to be a submucosal tumor of uncertain etiology, the cystic lesion was confirmed by MRI and EUS to be a cyst. Further evaluation determined that it originated from the superficial duodenal layer rather than the muscularis propria, making endoscopic resection a safe and feasible approach [15].

A key distinction between our case and previously reported nonampullary duodenal adenomas with cystic features lies in anatomical location and procedural complexity. In this case, the cystic lesion was directly adjacent to the ampullary adenoma, raising concerns about its origin, the potential for incomplete resection, and the risk of complications such as bleeding or perforation. In contrast, previous reports have described nonampullary duodenal adenomas with cystic components arising from various etiologies, including Brunner’s gland hyperplasia, which differ in malignant potential and endoscopic resectability [4,5].

Although this case demonstrates the effectiveness of en bloc endoscopic papillectomy in achieving complete resection with negative margins, several limitations must be acknowledged. First, while histopathological evaluation confirmed that the adjacent cystic lesion was a dilated glandular duct, its exact pathogenesis remains unclear. Further investigation is needed to determine whether such cystic lesions are incidental findings or hold clinical significance when found alongside ampullary adenomas. Second, as this is a single-case report, the generalizability of our findings is limited. Larger case series or prospective studies are necessary to validate our approach and establish clear selection criteria for endoscopic resection of ampullary adenomas with adjacent cystic lesions.

## Conclusions

This case demonstrates the feasibility and safety of en bloc endoscopic papillectomy for ampullary adenomas with adjacent cystic lesions. Despite the presence of a cystic lesion at the distal margin of the duodenal papillary tumor, thorough preoperative evaluation and meticulous treatment planning enabled successful en bloc resection with negative margins.
